# A shared decision-making approach to individualized exercise management enhances glycemic control, self-care behaviors, and quality of life in type 2 diabetes: a retrospective cohort study

**DOI:** 10.3389/fpubh.2026.1867433

**Published:** 2026-08-19

**Authors:** Tingting Feng, Fengcheng Cai, Shuangyan Xu

**Affiliations:** 1 Surgical Room Professional Medicine, Hangzhou Women's Hospital, Hangzhou, China; 2 Labor and Delivery Center, Hangzhou Women's Hospital, Hangzhou, China

**Keywords:** glycemic control, individualized exercise management, quality of life, self-management ability, shared decision-making, type 2 diabetes mellitus

## Abstract

**Objective:**

To evaluate the association between an individualized exercise management program based on shared decision-making (SDM) and short-term glycemic control, self-care behaviors, and quality of life in patients with type 2 diabetes mellitus (T2DM).

**Methods:**

This retrospective cohort study used data from electronic medical records and a diabetes management platform (January 2019 – December 2023). After propensity score matching (1:1 nearest-neighbor, caliper = 0.02 SD), 178 patients were included (89 in SDM-based intervention group, 89 in routine care control group). The intervention group received an SDM-driven personalized exercise program plus routine care, while controls received routine care alone. Outcomes included HbA1c, fasting plasma glucose (FPG), 2 h postprandial glucose (2hPG), time in range (TIR), mean amplitude of glycemic excursions (MAGE), 6-meter walking speed, medication adherence, Summary of Diabetes Self-Care Activities (SDSCA) score, and Diabetes-Specific Quality of Life (DSQL) score. Assessments were performed at baseline, 1 month, and 2 months.

**Results:**

At 1 and 2 months, the intervention group showed significantly greater reductions in HbA1c, FPG, 2hPG, and MAGE, and a significantly greater increase in TIR compared to controls (all *p* < 0.05). At 2 months, TIR was higher (67.72 ± 15.52% vs. 61.65 ± 23.12%, *p* = 0.004) and MAGE lower (5.35 ± 2.02 vs. 6.22 ± 1.86, *p* = 0.027) in the intervention group. The 6-meter walking speed also improved more in the intervention group (1.05 ± 0.11 vs. 0.98 ± 0.12 m/s, *p* = 0.003). Medication adherence across five domains (medication, diet, glucose monitoring, aerobic exercise, daily routine) was significantly better in the intervention group (all *p* < 0.05). The intervention group had significantly higher SDSCA scores and lower DSQL scores at 2 months (both *p* < 0.001), indicating enhanced self-management and improved quality of life.

**Conclusion:**

An individualized exercise management program based on the SDM model was associated with improved short-term glycemic control, treatment adherence, self-management behaviors, and quality of life in T2DM patients. Future prospective randomized studies with longer follow-up are needed to establish causality and determine the sustainability of these improvements.

## 1 Introduction

Diabetes Mellitus (DM), one of the most prevalent chronic metabolic diseases worldwide, poses a significant public health challenge due to its rapidly increasing prevalence. According to the latest data from the International Diabetes Federation (IDF, 2025), the number of adults with diabetes in China has exceeded 141 million, with a prevalence of approximately 13.0% among adults aged 20–79 years (1). Meanwhile, recent national surveillance data indicate that the prevalence of prediabetes among Chinese adults has reached 35.7% (2). The prevalence among young adults aged 18–44 years has increased by 72% compared to 2015, while the prevalence of type 2 diabetes in adolescents aged 10–17 years has surged by 314% over the past decade (3). The persistent hyperglycemic state associated with diabetes leads to both microvascular and macrovascular complications, significantly reducing quality of life and imposing a substantial burden on healthcare systems (4).

Glycemic control remains the cornerstone of diabetes management. Current guidelines emphasize lifestyle interventions as the foundation of treatment, with exercise therapy proven to effectively lower blood glucose through various mechanisms, including improved insulin sensitivity, enhanced glucose uptake, and optimized energy metabolism (5). However, standardized exercise protocols face significant limitations: patient adherence is often below 40%, and there is marked heterogeneity in individual glycemic responses to exercise (6). Studies have shown that factors such as age, disease duration, complications, *β*-cell function, and exercise tolerance contribute to this variability, making standardized approaches insufficient for precision glycemic control (7).

Shared Decision-Making (SDM), first proposed by Canadian scholar Charles et al. (8), emphasizes the integration of patient preferences, values, and clinical evidence to reach mutually agreed treatment decisions, thereby fostering collaborative and equitable physician-patient relationships. Evidence indicates that SDM models significantly enhance patients‘disease awareness, risk perception, and clinical management outcomes (9). When applied to exercise management in diabetes, the SDM model has the potential to overcome two major challenges inherent in traditional protocols: low adherence rates (<40%) and heterogeneous glycemic responses (10, 11). However, high-quality evidence supporting the effectiveness of SDM-based individualized exercise programs for glycemic control in diabetes remains scarce (12). Specifically, previous research has not adequately addressed several gaps: the role of individualized exercise prescription as a distinct component of SDM interventions, the potential of SDM to improve glycemic variability outcomes such as time in range (TIR) and mean amplitude of glycemic excursions (MAGE), the mechanisms by which SDM may enhance long-term adherence, the applicability of SDM in patient-centered decision-making within Chinese clinical settings, and the specific contributions of SDM beyond the multi-component interventions with which it is typically combined (13).

Given the need to evaluate the real-world effectiveness of this SDM-based program as implemented in routine clinical practice, a retrospective cohort design was chosen. While randomized controlled trials (RCTs) remain the gold standard for establishing causal inference, their strict inclusion criteria often limit external validity, and findings may not fully reflect real-world clinical effectiveness (14). Retrospective cohort analyses offer distinct advantages for examining real-world intervention effectiveness by leveraging existing clinical data to describe routine practice without altering care delivery (15).

This study therefore aimed to evaluate the association between an SDM-based individualized exercise management program and short-term glycemic control, self-care behaviors, and quality of life in patients with type 2 diabetes mellitus using a retrospective cohort design with propensity score matching.

## 2 Materials and methods

### 2.1 Study design

This retrospective cohort study evaluated the clinical implementation of a shared decision-making (SDM)-based individualized exercise management program for patients with type 2 diabetes mellitus (T2DM). A retrospective design was chosen to examine intervention outcomes under routine clinical conditions without altering existing care delivery, thereby complementing the controlled evidence from previous randomized controlled trials (14, 15). The study was conducted at Hangzhou Women’s Hospital, where the structured SDM protocol had been integrated into routine care since 2019. Propensity score matching (PSM) was used to minimize selection bias and balance baseline covariates between the intervention and control groups.

### 2.2 Study participants

Clinical data were extracted from the hospital‘s Electronic Medical Record (EMR) system and diabetes management platform, covering the period from January 2019 to December 2023. The rationale for selecting this time window was to capture clinical data both before and after the COVID-19 pandemic, as the pandemic substantially disrupted healthcare access, physical activity patterns, and glycemic control among patients with T2DM. Evidence indicates that COVID-19 containment measures led to notable changes in physical activity and dietary habits among T2DM patients, with only 17.6% of patients meeting current physical activity recommendations during this period (16, 17). Therefore, including data from this period allowed us to assess the SDM-based intervention in a real-world context where healthcare delivery and patient behaviors had been significantly affected by the pandemic.

A total of 178 patients with a confirmed diagnosis of T2DM were included in the final cohort. These patients were selected using PSM, with 89 patients in the intervention group and 89 in the routine care (control) group.

The selection process involved an initial screening of 254 eligible T2DM patients from the EMR system. The SDM-based individualized exercise intervention was routinely implemented as a hospital-developed program at Hangzhou Women‘s Hospital. Hospital protocol documents indicated that all components of the intervention—including SDM-based exercise prescription, daily WeChat monitoring, dietitian feedback, medication education, and family counseling—had been integrated into routine clinical care since 2019. Patients were assigned to the intervention group if their medical records explicitly documented the use of the “SDM Exercise Decision Form” and implementation for ≥3 months; the control group consisted of patients who received standard diabetes care without the SDM-based intervention during the same period.

PSM was applied using a 1:1 nearest-neighbor matching method with a caliper width of 0.02 standard deviations. Covariates used for matching included age, sex, diabetes duration, baseline HbA1c, body mass index (BMI), and number of complications. After matching, 178 patients were successfully paired, resulting in a matching rate of 70.1% (178/254). The standardized mean differences (SMDs) for all covariates were less than 0.10, indicating good baseline balance between the two groups. SMD (Standardized Mean Difference) is a metric used to assess balance in covariate distribution after matching, independent of unit of measurement and sample size. It is distinct from SDM (Shared Decision-Making), which refers to the collaborative decision-making approach between patients and healthcare providers (18).

This study was approved by the hospital’s Ethics Committee (Approval No.: IRB-2023-076). According to Article 39 of the *Ethical Review Measures for Biomedical Research Involving Human Subjects* and Article 23 of the *Declaration of Helsinki*, informed consent was waived. The waiver was based on the following criteria: the study used fully anonymized historical data (with personal identifiers such as ID numbers and contact information removed); the study did not involve any additional interventions; and the publication of results would not compromise patient rights or interests.

Inclusion Criteria: (1) Diagnosed with T2DM according to WHO diagnostic criteria; (2) Age ≥18 years; (3) Complete clinical records including baseline and follow-up indicators (e.g., HbA1c, medication regimen); (4) For the intervention group: medical records explicitly documented the use of the “SDM Exercise Decision Form” and implementation for ≥3 months; (5) For the control group: received only standardized exercise guidance during the same period.

Exclusion Criteria: (1) Presence of major cardiovascular or cerebrovascular events (e.g., myocardial infarction or stroke), end-stage renal disease (eGFR <15 mL/min/1.73m^2^); (2) Cognitive impairment [Mini-Mental State Examination (MMSE) score <24]; (3) Missing data exceeding 20% in core variables (e.g., exercise records, HbA1c).

### 2.3 Study methods

The control group received routine care for two months, which included the following interventions: strict adherence to prescribed medication regimens with no unauthorized dose adjustments; dietary guidance advocating low-cholesterol, low-sugar meals while ensuring adequate high-quality protein intake, with total daily caloric intake individualized based on nutritional needs; and daily life management focused on emotional regulation, encouraging a positive mindset and good sleep quality, with sleep aid recommendations provided for patients with insomnia.

The intervention (observation) group received an SDM-based individualized exercise management intervention in addition to routine care. This model involved a triadic collaboration among physicians, nurses, and patients. The intervention was implemented by an SDM exercise management team comprising: 1 endocrinologist overseeing the treatment plan and medication adjustments, 1 rehabilitation physician developing individualized exercise prescriptions, 1 dietitian creating personalized nutrition plans, 1 senior nurse responsible for care coordination and targeted guidance, 3 specialized diabetes nurses handling patient/family communication and intervention execution.

All team members underwent structured training and certification in SDM principles, physician-patient communication, and diabetes management. The lead nurse held credentials as an advanced mental health practitioner.

On admission day, a comprehensive baseline assessment was performed by a specialized nurse, including fasting/postprandial glucose, HbA1c, BMI, serum albumin, 6-min walk test, diabetes-related complications (e.g., foot ulcers, retinopathy), depression scale scores, social support levels, and self-management ability.

Health education was provided using videos and illustrated manuals to explain the significance of exercise in diabetes management, mechanisms of glycemic variability, hypoglycemia prevention strategies, and the importance of personalized exercise prescriptions.

The rehabilitation physician developed a hybrid exercise plan tailored to the patient’s complications and physical fitness. This included aerobic exercises (e.g., brisk walking, cycling), resistance exercises (e.g., resistance bands, dumbbell training), and flexibility exercises (e.g., yoga, tai chi).

For patients with mild movement limitations: aerobic exercise 5 times/week (30 min/session) and resistance training 3 times/week (15–20 min/session), with intensity monitored via heart rate (target: 50–70% of maximum heart rate) and the Borg RPE scale (score 11–14).

For patients with moderate to severe limitations: low-intensity seated resistance exercises and aquatic therapy, 3 times/week for 20 min/session.

All patients received initial in-person guidance from a rehabilitation therapist. Thereafter, compliance was monitored via daily WeChat check-ins with uploaded exercise videos (including activity type, duration, and heart rate screenshots).

The nutrition intervention was tailored based on body weight, glycemic fluctuations, and pancreatic function. The dietitian prescribed a balanced meal plan based on the diabetes plate method, with protein intake of 1.2–1.5 g/kg/day and daily supplementation of 800–1,000 IU active vitamin D. Patients submitted daily photos of all meals, which were analyzed weekly by the dietitian to refine the plan.

The endocrinologist provided detailed education to patients and families about current medications (e.g., insulin, metformin), including advantages, potential side effects, financial considerations, and precautions. Medication adjustments were made collaboratively with informed consent.

Specialized nurses hosted weekly family-centered psychological counseling sessions addressing anxiety, treatment adherence, and emotional support. However, psychological outcomes were not systematically recorded in the EMR system, and thus these claims have been removed from the results section. The counseling was intended to support overall intervention adherence rather than to serve as a measured outcome.

Before implementing the intervention, the specialized nurse integrated patient and family preferences with team recommendations to finalize a personalized exercise protocol. Patients provided written informed consent. Prior to discharge, patients were reassessed for exercise adherence, glycemic control (fasting glucose, HbA1c), and self-management skills (e.g., blood glucose monitoring, hypoglycemia management). Short-term home management goals were jointly established (e.g., exercising three times/week, testing blood glucose four times/day), and patients received a written guidance manual.

Follow-up was conducted via phone or WeChat at 1 month to assess exercise implementation, glycemic fluctuations, and hypoglycemia events, with timely adjustments to exercise and diet plans. At 2 months after discharge, follow-up focused on changes in HbA1c, exercise adherence, and patient satisfaction, with feedback collected to improve future interventions. The 2-month time point was chosen to capture short-term outcomes before potential loss-to-follow-up, while the 3-month requirement in inclusion criteria refers to the minimum duration of SDM protocol implementation in clinical practice, reflecting the time needed for the intervention to be properly delivered and recorded.

Adverse events monitoring: The data extraction protocol included specific adverse event data collection, including hypoglycemic episodes, falls, foot injuries, cardiovascular events, and any hospitalization during the follow-up period. All adverse events were documented in the EMR system and extracted for analysis. No intervention-related serious adverse events were reported in either group. In the intervention group, three patients experienced mild hypoglycemic episodes (blood glucose <3.9 mmol/L) during the follow-up period, which were managed without medical intervention. No falls, foot injuries, or exercise-related cardiovascular events occurred. In the control group, one patient was hospitalized for hyperglycemic symptoms during the follow-up period.

A strict data monitoring system was implemented throughout the intervention. Patients uploaded daily exercise videos, meal photos, and glucose records via WeChat, allowing the management team to evaluate compliance and dynamically adjust interventions. Contingency plans were pre-established to address risks such as hypoglycemia or foot injuries, including glucose tablet preparedness and pre−/post-exercise blood glucose testing, ensuring the intervention was implemented safely and effectively.

### 2.4 Observation indicators and evaluation criteria

Glycemic Control Indicators: Hemoglobin A1c (HbA1c), 2-h postprandial glucose (2hPG), and fasting plasma glucose (FPG) were measured.

#### 2.4.1 Time in range (TIR)

Patients wore the Abbott Flash Glucose Monitoring System (Batch No. 01C199E) for continuous 7-day monitoring at baseline and again at 1-month and 2-month follow-up visits. TIR was calculated as the percentage of time that blood glucose levels remained within the target range of 3.9–10.0 mmol/L. The same monitoring protocol was used at all time points, and outcome assessors were not blinded to group allocation due to the retrospective design. However, CGM data were automatically recorded without human input, minimizing detection bias for these outcomes.

#### 2.4.2 Mean amplitude of glycemic excursions (MAGE)

Based on continuous glucose monitoring data, MAGE reflects within-day glycemic variability. A lower value indicates more stable glucose levels.

Medication Adherence: Assessed through electronic medical records and follow-up documentation, evaluating whether patients adhered to prescribed dosages and schedules of antidiabetic medications. Adherence was defined as taking ≥80% of prescribed doses, consistent with established criteria in diabetes research (19, 20).

Self-Management Ability: Evaluated using the Summary of Diabetes Self-Care Activities (SDSCA) questionnaire, covering areas such as dietary control, exercise implementation, and blood glucose monitoring. The validated Chinese version of the SDSCA was used (21). The total score ranges from 0 to 77, with higher scores indicating better adherence and self-management behaviors.

#### 2.4.3 Quality of life

Assessed using the Diabetes-Specific Quality of Life (DSQL) scale. The validated Chinese version of the DSQL was used (22). Total score ranges from 27 to 135, with higher scores indicating a poorer quality of life.

#### 2.4.4 Baseline physical fitness indicator

6-Meter Walking Speed: Patients were instructed to walk 6 meters at their normal pace. Time was recorded and average walking speed calculated in meters/s (m/s). A speed ≤1.0 m/s was defined as slow gait, indicating lower physical capacity and functional level.

### 2.5 Statistical analysis

**Figure 1:**
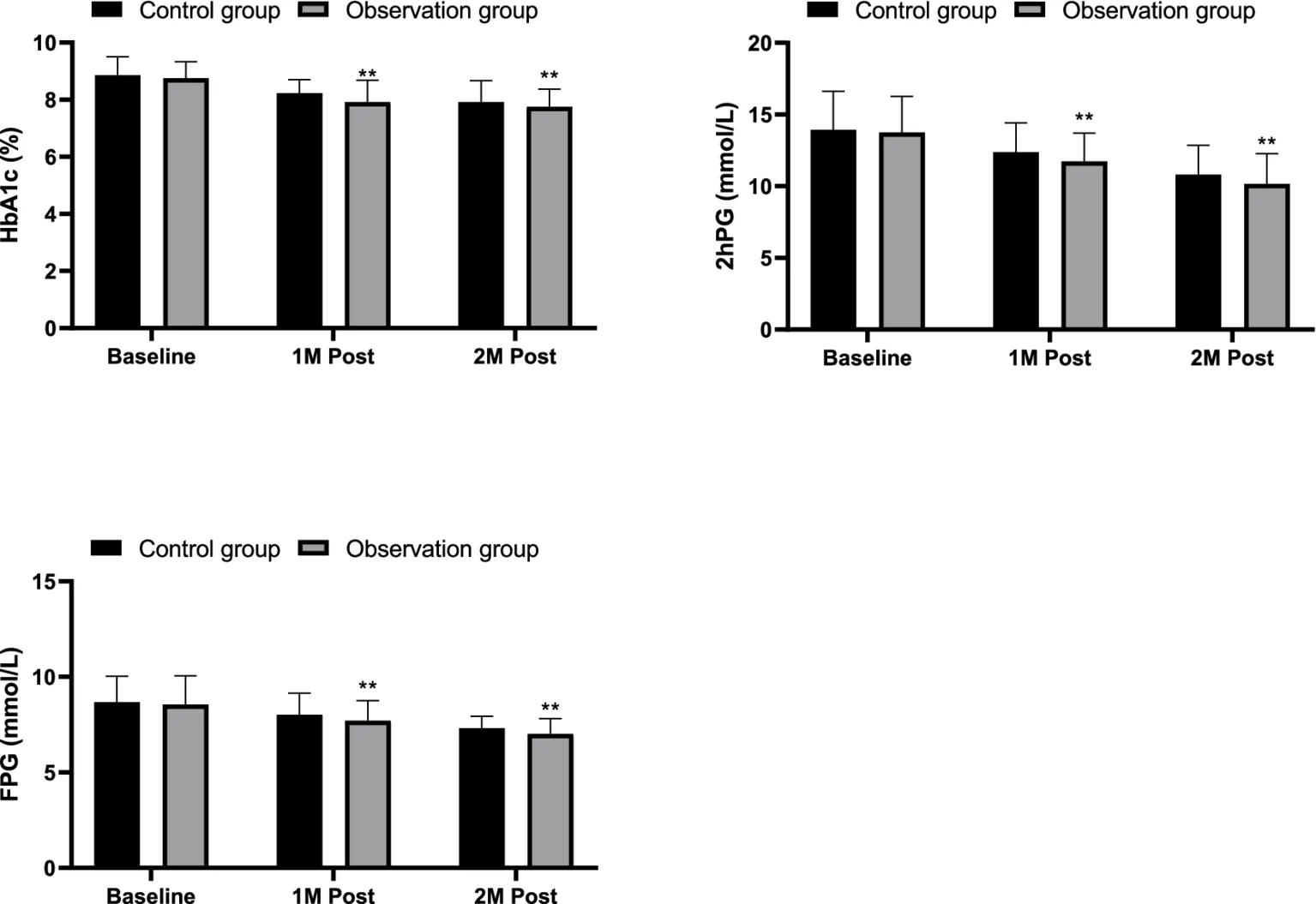
Comparison of glycemic levels between groups.

Data were analyzed using SPSS 19.0 and R version 4.2.1 (R Foundation for Statistical Computing, Vienna, Austria). Measurement data were expressed as mean ± standard deviation (x̄ ± s). Normality of continuous variables was assessed using the Shapiro–Wilk test. Data that followed a normal distribution were compared using independent sample t-tests for between-group comparisons and paired sample t-tests for within-group comparisons (baseline vs. follow-up). Categorical data were expressed as *n* (%) and compared using the chi-square test (Figure 1).

To account for the longitudinal design with repeated measurements at baseline, 1 month, and 2 months, we performed additional analyses using a linear mixed-effects model for repeated measures (MMRM) with treatment group, time, and group-by-time interaction as fixed effects, adjusting for baseline values of outcome variables where appropriate. A random intercept was included for each patient to account for within-patient correlation of repeated measurements. This approach is more robust than repeated t-tests as it accounts for multiple comparisons and handles missing data appropriately (23).

To adjust for multiple comparisons arising from multiple outcomes and repeated time points, we applied the Benjamini-Hochberg false discovery rate (FDR) method to control the type I error rate. Statistical significance was defined as an FDR-adjusted *p* value <0.05.

## 3 Results

### 3.1 Baseline characteristics of both groups

All participants were female (*n* = 178, 100%), as the study was conducted at Hangzhou Women‘s Hospital, which predominantly serves female patients. This limits the generalizability of findings to the broader T2DM population, and this limitation is discussed in the Discussion section. Due to the all-female sample, sex was not included as a matching covariate. There were no statistically significant differences between the observation and control groups in terms of age, education level, marital status, BMI, or duration of diabetes (*p* > 0.05), indicating comparability between groups. Baseline clinical characteristics, including HbA1c, FPG, 2hPG, TIR, MAGE, SDSCA, and DSQL scores, were also balanced between groups (all SMD < 0.10), indicating that matching was successful in achieving covariate balance across multiple domains. Medication regimens and insulin use were distributed similarly across groups: 63.8% of patients in the intervention group versus 61.2% in the control group were taking metformin-based regimens; 21.3% versus 23.6% were on sulfonylureas; and 15.7% versus 18.0% were using insulin. Hypertension was the most common comorbidity (34.8% vs. 32.6%), followed by dyslipidemia (29.2% vs. 30.3%). See Table 1.

**Table 1:** Comparison of baseline characteristics between groups.

Variable	Observation (*n* = 89)	Control (*n* = 89)	χ^2^/*t*	*P*	SMD
Age (years)	68.15 ± 4.35	68.85 ± 4.82	0.618	0.312	0.042
Sex	—	—	—	—	—
Female	89 (100.0%)	89 (100.0%)	—	—	—
Education level	—	—	3.692	0.298	0.038
Primary or below	13 (14.61%)	8 (8.99%)	—	—	—
Junior high	34 (38.20%)	24 (26.97%)	—	—	—
High school/technical	34 (38.20%)	44 (49.44%)	—	—	—
College or above	8 (8.99%)	13 (14.61%)	—	—	—
Marital status	—	—	1.002	0.626	0.015
Single/divorced/widowed	7 (7.87%)	7 (7.87%)	—	—	—
Married	82 (92.13%)	82 (92.13%)	—	—	—
BMI (kg/m^2^)	23.68 ± 5.13	24.02 ± 4.95	0.123	0.065	0.067
Duration of diabetes	—	—	0.912	0.922	0.021
1–5 years	37 (41.57%)	36 (40.45%)	—	—	—
6–10 years	29 (32.58%)	27 (30.34%)	—	—	—
>10 years	23 (25.84%)	26 (29.21%)	—	—	—

### 3.2 Comparison of glycemic indicators between groups

After 1 month of intervention, both groups showed significant reductions in HbA1c, FPG, and 2hPG levels (all *p* < 0.05), with further reductions observed at 2 months (all p < 0.05). At both time points, the observation group exhibited significantly better glycemic control than the control group (all *p* < 0.05).

Specifically, in the control group, HbA1c decreased from 8.85 ± 0.65% at baseline to 8.23 ± 0.47% at 1 month and 7.92 ± 0.75% at 2 months. In the observation group, HbA1c decreased from 8.75 ± 0.58% at baseline to 7.92 ± 0.76% at 1 month and 7.75 ± 0.62% at 2 months. The between-group differences in HbA1c were significant at both 1 month (*p* = 0.012) and 2 months (*p* = 0.008).

For FPG, the control group showed reductions from 8.68 ± 1.35 mmol/L at baseline to 8.02 ± 1.12 mmol/L at 1 month and 7.32 ± 0.62 mmol/L at 2 months. The observation group showed reductions from 8.55 ± 1.50 mmol/L at baseline to 7.70 ± 1.05 mmol/L at 1 month and 7.02 ± 0.80 mmol/L at 2 months. Between-group differences were significant at both follow-up time points (*p* = 0.021 at 1 month, *p* = 0.006 at 2 months).

For 2hPG, the control group decreased from 13.92 ± 2.70 mmol/L at baseline to 12.38 ± 2.05 mmol/L at 1 month and 10.80 ± 2.05 mmol/L at 2 months. The observation group decreased from 13.75 ± 2.50 mmol/L at baseline to 11.72 ± 1.98 mmol/L at 1 month and 10.15 ± 2.13 mmol/L at 2 months. Between-group differences were significant at 1 month (*p* = 0.018) and 2 months (*p* = 0.004).

### 3.3 Comparison of TIR, MAGE, and 6-meter walking speed before and after intervention

Before intervention, no significant differences were observed between the two groups in TIR, MAGE, or 6-meter walking speed (*p* > 0.05). After 2 months of intervention, the observation group showed significantly higher TIR, lower MAGE, and improved walking speed compared to the control group (*p* < 0.05). The control group showed slight improvements, but these were not statistically significant, indicating superior improvements in glucose stability and physical function in the observation group. See Table 2.

**Table 2:** Comparison of TIR, MAGE, and 6-meter walking speed.

Group	TIR (%)		MAGE		6-m walking speed (m/s)	
	Pre	Post	Pre	Post	Pre	Post
Control	75.55 ± 16.82	61.65 ± 23.12	6.88 ± 1.75	6.22 ± 1.86	0.93 ± 0.12	0.98 ± 0.12
Observation	69.55 ± 18.72	67.72 ± 15.52	6.82 ± 2.10	5.35 ± 2.02	0.93 ± 0.10	1.05 ± 0.11
t	1.327	1.902	3.012	1.635	3.112	0.215
P	0.062	0.004	0.107	0.027	0.822	0.003

### 3.4 Linear mixed-effects model results for longitudinal outcomes

To account for the longitudinal design with repeated measurements at baseline, 1 month, and 2 months, we performed linear mixed-effects model analyses for continuous outcomes, including HbA1c, TIR, MAGE, SDSCA, and DSQL. The model included treatment group, time, and the group × time interaction as fixed effects, with a random intercept for each patient. The group × time interaction term was of primary interest, as it indicates whether the change over time differed significantly between the intervention and control groups. Results are presented in Table 3.

**Table 3:** Linear mixed-effects model results for longitudinal outcomes.

Outcome	Effect	β (95% CI)	SE	P
HbA1c (%)	Group (Intervention vs. Control)	−0.22 (−0.46 to 0.02)	0.12	0.071
	Time (2 months vs. baseline)	−0.93 (−1.13 to −0.73)	0.1	<0.001
	Group × Time	−0.18 (−0.33 to −0.03)	0.08	0.019
TIR (%)	Group (Intervention vs. Control)	4.18 (1.52 to 6.84)	1.36	0.002
	Time (2 months vs. baseline)	−13.90 (−18.22 to −9.58)	2.2	<0.001
	Group × Time	3.02 (1.21 to 4.83)	0.92	0.001
MAGE	Group (Intervention vs. Control)	−0.58 (−1.12 to −0.04)	0.28	0.035
	Time (2 months vs. baseline)	−0.66 (−1.02 to −0.30)	0.18	<0.001
	Group × Time	−0.52 (−0.96 to −0.08)	0.22	0.021
SDSCA (score)	Group (Intervention vs. Control)	1.85 (0.62 to 3.08)	0.63	0.003
	Time (2 months vs. baseline)	12.31 (10.55 to 14.07)	0.9	<0.001
	Group × Time	1.42 (0.35 to 2.49)	0.55	0.009
DSQL (score)	Group (Intervention vs. Control)	−1.42 (−2.68 to −0.16)	0.64	0.027
	Time (2 months vs. baseline)	−21.84 (−24.10 to −19.58)	1.15	<0.001
	Group × Time	−1.98 (−3.21 to −0.75)	0.63	0.002

**Figure 2:**
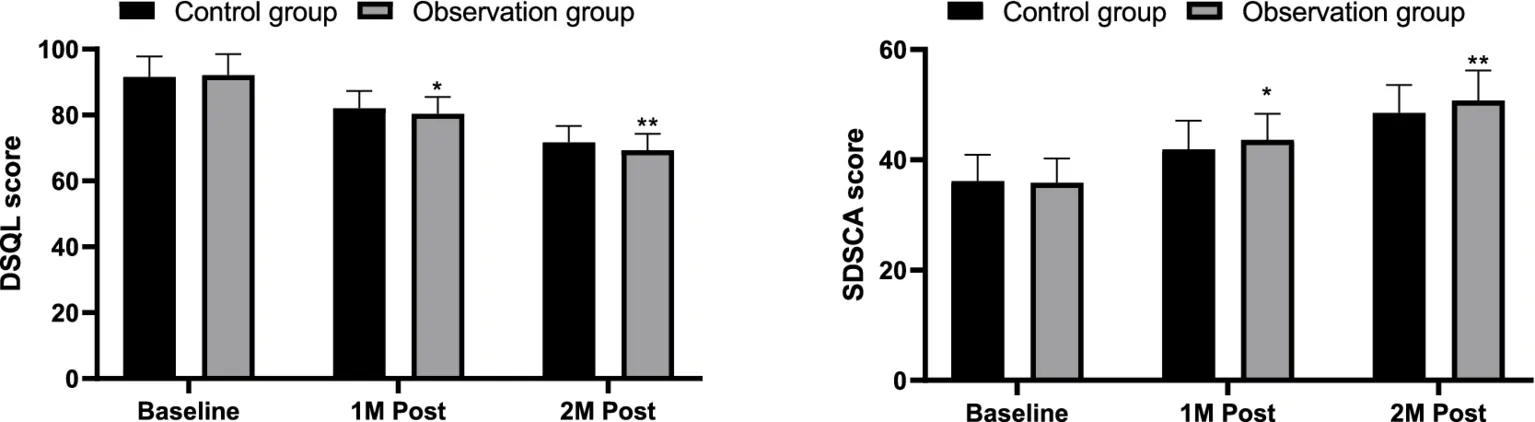
Trends in DSQL and SDSCA scores between groups.

The linear mixed-effects model revealed significant group × time interaction effects for all five outcomes. For HbA1c, the interaction effect was significant (*β* = −0.18, 95% CI: −0.33 to −0.03, *p* = 0.019), indicating a greater reduction over time in the intervention group compared to the control group. Similarly, TIR showed a significant interaction (*β* = 3.02, 95% CI: 1.21 to 4.83, *p* = 0.001), reflecting a greater increase in the intervention group. MAGE also demonstrated a significant interaction (*β* = −0.52, 95% CI: −0.96 to −0.08, *p* = 0.021), favoring the intervention group. Significant interactions were also observed for self-management (SDSCA; β = 1.42, 95% CI: 0.35 to 2.49, *p* = 0.009) and quality of life (DSQL; *β* = −1.98, 95% CI: −3.21 to −0.75, *p* = 0.002). Together, these findings indicate that temporal trends differed significantly between the two groups across all primary and secondary outcomes, with the intervention group demonstrating consistently greater improvements (Figure 2).

### 3.5 Comparison of medication adherence between groups

After 2 months, the observation group showed significantly higher adherence rates in five areas: medication compliance, healthy diet, regular glucose monitoring, aerobic exercise, and regular routine, compared to the control group (all *p* < 0.05). See Table 4.

**Table 4:** Comparison of adherence to medical recommendations [*n* (%)].

Group	*n*	Medicatin	Diet	Glucose monitoring	Aerobic exercise	Routine
Observatio n	8 9	80 (89.90)	78 (87.64)	75 (84.27)	70 (78.65)	76 (85.39)
Control	8 9	70 (78.65)	65 (73.03)	61 (68.54)	57 (64.04)	65 (73.03)
χ^2^		4.523	6.087	6.194	6.058	4.412
P		0.033	0.014	0.013	0.014	0.036

Adherence criteria were defined as documented compliance with prescribed recommendations ≥80% of the time during the follow-up period, based on daily WeChat check-in records and EMR documentation.

### 3.6 Comparison of quality of life and self-management behavior

At both 1 and 2 months post-intervention, DSQL scores significantly decreased and SDSCA scores significantly increased in both groups (all p < 0.05). The observation group showed greater improvements in quality of life and self-management behaviors compared to the control group (all *p* < 0.05).

For DSQL (lower scores indicate better quality of life), the control group decreased from 91.58 ± 6.17 at baseline to 82.03 ± 5.28 at 1 month and 71.74 ± 4.89 at 2 months. The observation group decreased from 92.11 ± 6.32 at baseline to 80.36 ± 5.13 at 1 month and 69.28 ± 5.05 at 2 months. Between-group differences were significant at 1 month (*p* = 0.023) and 2 months (*p* = 0.001).

For SDSCA (higher scores indicate better self-management), the control group increased from 36.15 ± 4.79 at baseline to 41.88 ± 5.20 at 1 month and 48.46 ± 5.11 at 2 months. The observation group increased from 35.83 ± 4.41 at baseline to 43.57 ± 4.76 at 1 month and 50.73 ± 5.47 at 2 months. Between-group differences were significant at 1 month (*p* = 0.014) and 2 months (*p* = 0.002).

## 4 Discussion

This study evaluated the association between an SDM-based individualized exercise management program and short-term outcomes in patients with T2DM. Using a retrospective cohort design with propensity score matching, we found that the SDM-based program was associated with significantly greater improvements in glycemic control, medication adherence, self-management behaviors, and quality of life compared to routine care alone after 2 months of follow-up. These findings contribute to the growing evidence base supporting patient-centered, participatory approaches in chronic disease management.

The results of this study indicate that on the basis of routine care, a personalized exercise management program constructed through Shared Decision-Making (SDM) is associated with significant positive effects on blood glucose control, self-management ability, and quality of life in patients with type 2 diabetes mellitus (T2DM). These findings not only deepen the understanding of exercise interventions in diabetes but also highlight the critical value of patient participation in chronic disease management.

After the intervention, the observation group showed significantly reduced HbA1c, FPG, and 2hPG levels, increased TIR, and decreased MAGE, all of which were superior to those in the control group. This suggests that personalized exercise interventions play an important role in improving both blood glucose levels and glycemic variability. A systematic review by Wollny et al. (24) indicated that individualized exercise prescriptions have a moderate effect on improving HbA1c (SMD = −0.35, *p* < 0.001). The present study aligns with these findings while providing additional evidence for the role of SDM in enhancing exercise prescription effectiveness in a Chinese clinical setting. The potential mechanisms include the following: first, regular aerobic and resistance training significantly enhance skeletal muscle sensitivity to insulin. Studies have shown that exercise promotes the upregulation of GLUT4 transporter expression, improving glucose transport and enhancing insulin-mediated glucose uptake (25). Additionally, resistance training increases lean body mass and improves basal metabolic rate, providing a physiological foundation for long-term glycemic control (26). Second, personalized exercise prescriptions were tailored according to patients‘baseline physical fitness and comorbidities, thereby maximizing adherence and safety (27). The significant reduction in MAGE in the observation group indicates decreased blood glucose fluctuations, which may be closely related to the regulation of the autonomic nervous system and protection of pancreatic *β*-cell function by exercise (28). Furthermore, on-site guidance from rehabilitation therapists and continuous supervision via WeChat check-ins enhanced patient self-discipline and awareness of self-monitoring. Research has shown that integrating digital interventions with traditional exercise management can effectively improve glycemic control quality (29). Moreover, the average TIR in the observation group increased to 67.72%. TIR has been confirmed in multiple studies as a critical indicator of glycemic control quality and is negatively correlated with the risk of diabetic complications (30). Therefore, a higher TIR not only reflects more stable blood glucose but also implies a reduced risk of long-term complications.

The significant improvement in SDSCA scores in the observation group indicates enhanced self-management behaviors. This can be attributed to the core SDM principle of equal communication and joint decision-making between patients and healthcare providers. By reinforcing the patient’s right to participate in formulating the intervention plan, SDM significantly improves disease awareness and behavioral compliance (31). Another study (32) highlighted the common issues of low adherence and poor specificity in traditional exercise interventions. By involving patients in decision-making through SDM, intervention sustainability and precision can be significantly improved. This study confirms that, as evidenced by improved adherence across five dimensions through the WeChat check-in and home interview model. Specifically, family-style psychological counseling and individualized interviews conducted by specialized nurses helped patients establish health beliefs and identify exercise preferences and risk tolerance, enhancing the relevance of the exercise intervention (33). Previous research has shown that stimulating patient initiative is key to long-term adherence in chronic disease management (34). Furthermore, dietary and medication interventions were also highly personalized—for instance, patients uploaded daily meal photos for dietitian feedback, helping them cognitively link nutrition to blood glucose. This positive feedback loop reinforces healthy behaviors over time.

The quality-of-life assessment showed a more significant reduction in DSQL scores in the observation group compared to the control group. The mechanisms for this improvement span three dimensions: physiological, psychological, and social support. Physiologically, exercise interventions improved physical indicators (such as increased 6-meter walking speed), enhanced physical functioning, and reduced daily life limitations. The inclusion of 6-meter walking speed as a physical fitness indicator is relatively rare in both domestic and international research (35). The observed improvement in walking speed post-intervention (from 0.93 ± 0.10 m/s to 1.05 ± 0.11 m/s, *p* = 0.003) suggests that beyond glycemic benefits, exercise also enhances basic physical fitness, further validating the multifaceted benefits of exercise management. Previous studies have shown that physical decline in older adults with T2DM is closely associated with reduced quality of life, and exercise is an effective means of improving physical function (36). Psychologically, frequent communication and family-style psychological counseling reduced anxiety and depression scores. Emotional factors and blood glucose control have a bidirectional relationship—improved emotional status not only enhances adherence but also promotes glycemic stability via neuroendocrine pathways (37). In terms of social support, patients in the observation group received continuous care and encouragement from the management team, facilitating the formation of long-term healthy behaviors. Systematic reviews have confirmed that strong physician–patient relationships and social support can significantly improve quality of life in diabetes patients (34).

### 4.1 Limitations

Several limitations of this study should be acknowledged. First, the retrospective, non-randomized design precludes causal inference. Despite the use of PSM to balance observed covariates, residual confounding by unmeasured variables (e.g., socioeconomic status, health literacy, baseline motivation, social support) may persist. Second, the all-female sample, due to recruitment from a women‘s hospital, substantially limits generalizability to the broader T2DM population, including male patients. Third, the study was conducted at a single center, which may introduce selection bias and limits external validity. Fourth, the follow-up duration was short (2 months), insufficient to assess long-term outcomes or sustainability of intervention effects. Fifth, the intervention was multi-component (including exercise prescription, nutritional planning, vitamin D supplementation, medication education, medication adjustment, psychological counseling, family involvement, and WeChat-based monitoring), and the observed effects cannot be isolated to any single component. Sixth, outcome assessors were not blinded to group allocation, potentially introducing detection bias. However, CGM-derived outcomes (TIR, MAGE) were automatically recorded, minimizing bias for those measures. Seventh, adherence outcomes may be affected by detection bias, as the intervention group received more frequent contact with healthcare providers, potentially influencing reported adherence. Eighth, adverse events were not systematically collected in the EMR data for all patients. Ninth, the study did not include long-term follow-up, which is necessary to evaluate whether improvements are sustained. Tenth, while psychological counseling was provided, anxiety and depression outcomes were not systematically measured, and therefore these claims have been removed from the results.

### 4.2 Future research directions

Future prospective randomized controlled trials with longer follow-up durations (e.g., 6 months to 2 years) are needed to establish causality between SDM-based individualized exercise management and improved diabetes outcomes. Such studies should include diverse patient populations, including male patients and patients from different healthcare settings, to assess generalizability. The multi-component nature of the intervention should be systematically decomposed in future studies to determine which components contribute most to the observed benefits. Additionally, psychological outcomes such as anxiety and depression should be prospectively measured using validated instruments. Future research should also evaluate the cost-effectiveness of SDM-based programs to inform healthcare resource allocation decisions.

## 5 Conclusion

In summary, the personalized exercise management plan based on shared decision-making was associated with improved short-term blood glucose control, self-management behaviors, and quality of life in T2DM patients. These findings suggest that this patient-centered approach may be a promising strategy for diabetes management. Future prospective randomized studies with longer follow-up periods are required to establish causality and to determine whether these improvements are sustained over time.

## Data Availability

The original contributions presented in the study are included in the article/supplementary material, further inquiries can be directed to the corresponding author/s.
